# Lessons from Four Years (2021–2024) of *Klebsiella Pneumoniae* Resistance Surveillance Epidemiological Trends in a Romanian Intensive Care Unit

**DOI:** 10.3390/antibiotics14080825

**Published:** 2025-08-12

**Authors:** Mihai Sava, Bogdan Ioan Vintila, Alina Simona Bereanu, Anca Maria Fratila, Ioana Roxana Codru

**Affiliations:** 1 Faculty of Medicine, Lucian Blaga University of Sibiu, 550169 Sibiu, Romania; mihai.sava@ulbsibiu.ro (M.S.); anca.fratila@ulbsibiu.ro (A.M.F.); ioanaroxana.bera@ulbsibiu.ro (I.R.C.); 2 County Clinical Emergency Hospital of Sibiu, 550245 Sibiu, Romania

**Keywords:** *Klebsiella pneumoniae*, ESKAPE, epidemiological surveillance, antibiotic resistance, intensive care unit, carbapenem-resistant enterobacteriaceae, MDR/XDR/PDR

## Abstract

**Background:** *Klebsiella pneumoniae* represents a major cause of healthcare-associated infections in intensive care units, with resistance profiles ranging from multidrug-resistant to extensively drug-resistant and pandrug-resistant. Critically ill patients, who often require invasive devices and prolonged antibiotic therapy, are especially vulnerable to colonization and infection by these strains. Surveillance data on resistance trends and specimen-specific patterns in Romanian intensive care units (ICUs) remain limited. **Methods:** We conducted a four-year surveillance study (2021–2024) in a tertiary Romanian ICU, analyzing *K. pneumoniae* isolates collected from diverse clinical specimens. Resistance phenotypes were classified as MDR, XDR, PDR, or susceptible based on standard definitions. Trends over time were assessed using Cramér’s V and correspondence analysis, while stratification by specimen type evaluated associations between anatomical site and resistance profiles. **Results:** A total of 254 *K. pneumoniae* isolates were analyzed. MDR strains predominated in 2021 and 2022 but sharply declined by 2024 (from 80% to 8.3%). In parallel, XDR and PDR phenotypes increased substantially, indicating a shift toward more complex resistance profiles. A significant temporal association was found (Cramér’s V = 0.43), with 2024 marked by a sharp decline in MDR isolates and a predominance of XDR and PDR phenotypes, reflecting an advanced resistance profile. Specimen-type analysis showed tracheal aspirates as the main reservoir for resistant strains, followed by urine and blood cultures, with a weaker but meaningful association (Cramér’s V = 0.24). **Conclusions:** These findings reveal a change in resistance patterns in ICU-acquired *K. pneumoniae* infections, with MDR strains being displaced by XDR and PDR phenotypes. These findings highlight the urgent need for time- and specimen-informed resistance monitoring and adaptive antimicrobial stewardship. Without targeted interventions, gains made in controlling MDR strains risk being rapidly eclipsed by the spread of highly resistant organisms.

## 1. Introduction

*Klebsiella pneumoniae* is a Gram-negative, encapsulated, non-motile, rod-shaped bacterium and is one of the leading causes of healthcare-associated infections, particularly in intensive care units. *Klebsiella pneumoniae*, a member of the Enterobacteriaceae family, is part of the normal gastrointestinal microbiota found in healthy humans and animals. However, within the healthcare system, it is an opportunistic pathogen commonly associated with ventilator-associated pneumonia, postoperative infections, and sepsis and is responsible for about one-third of all Gram-negative infections in hospitals. With the rise in antimicrobial resistance, *K. pneumoniae* has become recognized for its role in infections that are difficult to treat. Also, it is part of the ESKAPE group of multidrug-resistant organisms, which are recognized for their ability to evade the effects of antimicrobial therapy. Several factors, including capsular polysaccharides, lipopolysaccharides, siderophores such as aerobactin, and fimbriae, contribute to the virulence of this bacterium. *K. pneumoniae* primarily develops multidrug resistance through the production of β-lactamase enzymes. These include enzymes from Ambler class A (*Klebsiella pneumoniae* carbapenemase, KPC), class B (metallo-β-lactamases, MBLs), and class D (oxacillinases, OXA-48-like). These characteristics, combined with the virulence and resistance of Klebsiella pneumoniae, create significant challenges in clinical settings, particularly for vulnerable patients admitted to the intensive care unit [1,2,3,4,5,6,7,8].

Risk factors for developing resistant *Klebsiella pneumoniae* infections are prolonged intensive care unit stay, invasive medical therapies and monitoring devices, antibiotic misuse, high-risk surgery, and chronic illnesses such as malignancies, chronic obstructive pulmonary disease, and diabetes. Managing infections in critically ill patients is challenging due to their complex physiology and increased vulnerability. Among a weak immune system, invasive interventions (mechanical ventilation and central lines), and organ dysfunctions, the critically ill patient poses changes in pharmacokinetics that complicate dosing strategies for antibiotics [4,9,10,11,12,13].

The global emergence of carbapenemase-producing *Klebsiella pneumoniae* is a significant concern and has become a major public health issue due to its increasing prevalence worldwide. In Eastern Europe, particularly in Romania, data on the resistance patterns and epidemiological trends of *K. pneumoniae* remain limited, despite increasing clinical relevance. In Eastern, Southern, and Central Europe, there is a high rate of multidrug-resistant *K. pneumoniae*. Among these countries, Romania reports an increasing number of resistant *K. pneumoniae* strains isolated from invasive infections each year. In a study conducted in our department, *Klebsiella pneumoniae* demonstrated the highest detection frequency in the biofilm extracted from the endotracheal canula of patients diagnosed with ventilator-associated pneumonia. *Klebsiella pneumoniae’s* ability to form biofilms on both biological matter and medical devices is an important virulence trait. These biofilms protect bacterial communities from antibiotics and the host immune response, making infections more challenging to treat and facilitating their spread, posing another challenge to patient care and safety [14,15,16,17].

Epidemiological studies have identified two main pathways for the transmission of resistant Klebsiella pneumoniae. The first is direct patient-to-patient spread, which healthcare workers often facilitate, accounting for approximately 68% of transmissions in intensive care unit settings. The second pathway involves infections that arise from environmental reservoirs. Additionally, several independent risk factors can increase the likelihood of acquiring resistant *Klebsiella pneumoniae*. These include extended stay in the ICU, the use of invasive medical devices, and prolonged exposure to antibiotics. To address these risk factors and transmission pathways, it is clear that implementing measures is essential. Surveillance and containment strategies are essential to limiting the spread of multidrug-resistant *Klebsiella pneumoniae* and combating antibiotic resistance, especially in high-risk hospital settings. Measures that include screening newly admitted patients, microbiological analysis for clinically suspected infections, patient isolation, molecular typing, antibiotic susceptibility profiling, and surveillance of hospital environments and surfaces enable the identification of contaminated surfaces and colonized or infected patients, supporting prompt infection control interventions. These interventions include training staff, strict adherence to hand hygiene guidelines, environmental disinfection, proper medical waste management, and safe handling of patient secretions. Additionally, it is essential to regularly clean and disinfect surfaces and furniture, systematically monitor and report healthcare-associated infections, and adhere to protocols for infection prevention during invasive procedures. Structural interventions, such as configuring ICUs into smaller wards, have also been shown to help contain infections by reducing cross-transmissions. A recent study concluded that patients carrying resistant *Klebsiella pneumonia* who were hospitalized in a separate, small ward for the entire study period did not spread the bacteria to the other critically ill patients [18,19,20,21,22,23].

The aim of this study was to describe the epidemiological trends and antimicrobial resistance profiles of *Klebsiella pneumoniae* isolates collected over a four-year period (2021–2024) in a Romanian ICU in order to provide information to inform infection control strategies, guide empirical treatment, and enhance local and regional efforts in antimicrobial stewardship.

## 2. Results

This research evaluated resistance patterns over time to analyze temporal trends and detect changes in the prevalence of multidrug-resistant strains. Additionally, we investigated variations in resistance profiles according to the type of sample collected (e.g., blood, urine, tracheal aspirate, etc.). The primary outcome was the annual distribution of resistance categories, specifically MDR, XDR, PDR, and non-resistant isolates.

**Figure 1:**
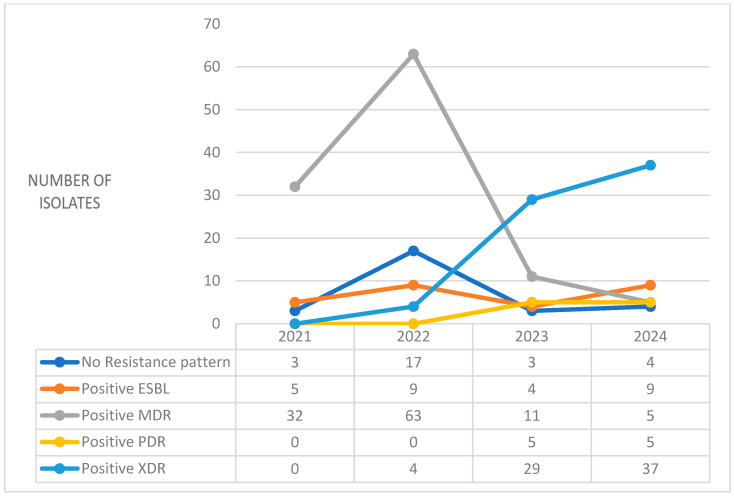
Annual distribution and resistance patterns of Klebsiella pneumoniae (2021–2024).

Figure 1 presents the annual distribution of resistance patterns observed in *Klebsiella pneumoniae* isolates from 2021 to 2024. A notable trend is the significant reduction in multidrug-resistant isolates, which decreased from 32 cases in 2021 to 5 cases in 2024. In contrast, the number of extensively drug-resistant isolates increased significantly, rising from 4 in 2022 to 37 in 2024, indicating a shift in the complexity of resistance. The number of isolates with no resistance pattern also varied, with the highest count observed in 2022. Extended-spectrum beta-lactamase (ESBL)-producing isolates remained relatively consistent across the years, while pandrug-resistant cases emerged in 2023 and 2024.

**Figure 2:**
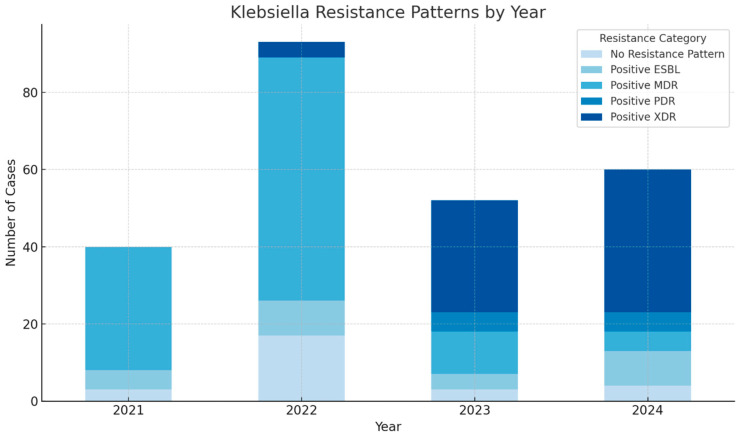
Annual distribution of Klebsiella pneumoniae resistance patterns (2021–2024).

The annual distribution of antimicrobial resistance categories in *Klebsiella pneumoniae* isolates from ICU patients between 2021 and 2024 is illustrated in Figure 2.

**Figure 3:**
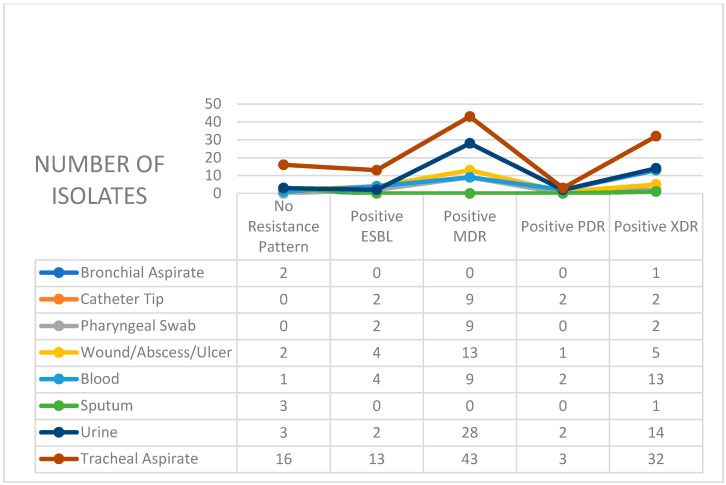
Distribution of Klebsiella pneumoniae resistance patterns by specimen type in ICU patients (2021–2024).

Figure 3 presents the distribution of resistance patterns in *Klebsiella pneumoniae* isolates, categorized by the type of specimen collected. The tracheal aspirates represent the most frequent specimen type from which *Klebsiella pneumoniae* was isolated, showing the highest counts of MDR and XDR isolates. Urine and blood specimens also showed significant resistance, particularly with elevated XDR frequencies. Catheter tips, wounds/abscesses, and pharyngeal swabs exhibited moderate resistance levels, whereas bronchial aspirates showed minimal resistance, with one XDR isolate and no MDR or PDR cases.

**Figure 4:**
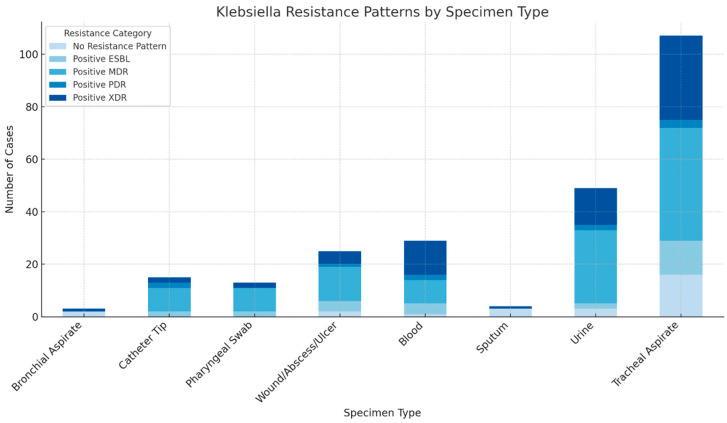
Distribution of Klebsiella pneumoniae resistance patterns by specimen type in ICU patients (2021–2024).

Figure 4 displays the distribution of *Klebsiella pneumoniae* resistance profiles by specimen type. Tracheal aspirates showed the highest overall resistance burden, with particularly elevated numbers of MDR and XDR strains. Urine and blood cultures also significantly contributed to the pool of resistant isolates. At the same time, other specimen types, such as sputum, bronchial aspirates, and swabs, presented fewer cases and a more limited resistance spectrum.

The results of our statistical analysis revealed clear and statistically significant temporal and specimen-specific variations in *Klebsiella pneumoniae* antimicrobial resistance patterns within our ICU setting over the study period (2021–2024).

The distribution of resistance profiles across the years demonstrated a highly significant change (Chi-square = 137.36, *p* ≈ 2.06 × 10^−20^), strongly suggesting that resistance patterns have not remained static over time. Notably, the associated effect size, as measured by Cramér’s V (0.43), indicates a moderate to strong relationship between the year of isolation and the identified resistance pattern. This finding suggests significant year-on-year changes in the local microbiological landscape.

In parallel, our analysis of specimen-type-specific resistance patterns also revealed statistically significant variability (Chi-square = 87.97, *p* = 0.0024). While the degree of association was less pronounced (Cramér’s V = 0.24), the result still indicates a weak to moderate association between the type of clinical specimen and the observed resistance pattern.

**Figure 5:**
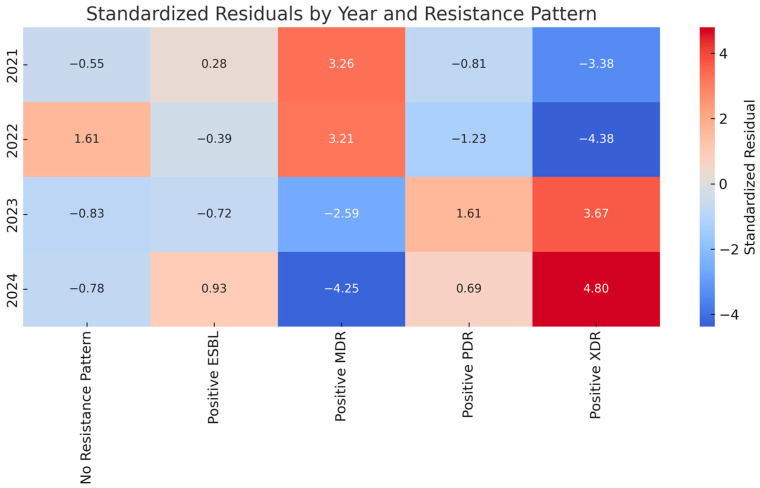
Heatmap of standardized residuals from Chi-square analysis of Klebsiella pneumoniae resistance patterns by year.

Figure 5 presents the standardized residuals from the Chi-square analysis, highlighting which resistance categories deviated most significantly from the expected values across the years. Positive residuals indicate an observed count higher than expected, while negative values suggest underrepresentation.

In 2021 and 2022, MDR isolates were significantly overrepresented, as indicated by residuals exceeding +3. In contrast, 2023 and especially 2024 saw substantial underrepresentation of MDR (residuals of −2.59 and −4.25), coinciding with a sharp rise in XDR strains (residuals of +3.67 and +4.80). Similarly, PDR patterns were moderately overrepresented in the last two years, while “No Resistance Pattern” values fluctuated without reaching critical thresholds. The heatmap visualization of the standardized residuals, outlined in Figure 3, provides a clear view of which resistance patterns were over- or underrepresented in each year. Red shades highlight categories with significantly higher-than-expected cases, while blue shades represent underrepresentation. Values near 0 indicate observed counts that are close to the expected values.

**Figure 6:**
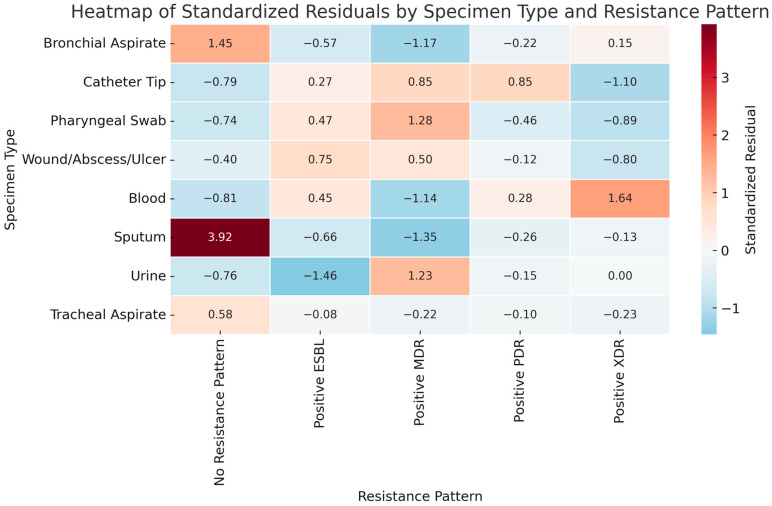
Heatmap of standardized residuals from Chi-square analysis of Klebsiella pneumoniae resistance patterns by specimen type.

Figure 6 illustrates the standardized residuals from the Chi-square analysis of resistance patterns across different specimen types. The most significant positive deviation was observed in bronchial aspirates and sputum within the “No Resistance Pattern” category, indicating a markedly higher-than-expected frequency of non-resistant isolates in these respiratory samples. In contrast, catheter tips and pharyngeal swabs showed a notable overrepresentation of MDR and PDR strains. Blood cultures were particularly associated with XDR isolates, highlighting their critical clinical implications. Tracheal aspirates exhibited a more balanced pattern with only mild deviations.

**Figure 7:**
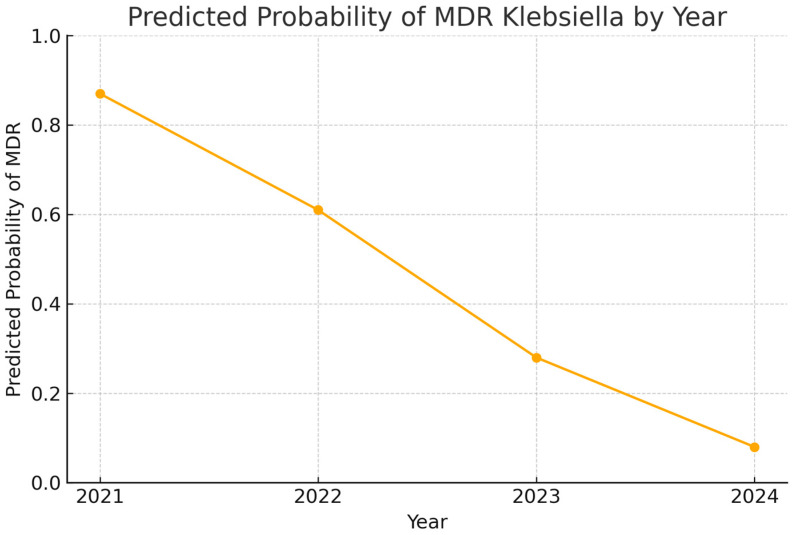
Predicted decline in probability of MDR Klebsiella pneumoniae infections from 2021 to 2024.

Statistical analyses revealed significant differences in the resistance patterns of Klebsiella isolates both across years and specimen types. To further investigate the dynamics observed in the resistance patterns over time, we employed logistic regression analysis to model the probability of an MDR *Klebsiella pneumoniae* infection as a function of the year (Figure 7). The model revealed a statistically significant negative association between year and the likelihood of MDR, with a year coefficient of −1.45 (*p* < 0.0001). This suggests that for each successive year from 2021 to 2024, the log-odds of identifying an MDR strain decreased significantly. The model showed a pseudo-R^2^ value of 0.261, indicating a moderate fit, and the log-likelihood ratio test yielded a highly significant *p*-value of 5.97 × 10^−21^, reinforcing the strength of this downward trend.

Supporting this regression finding, a two-proportion z-test comparing MDR prevalence between 2021 and 2024 demonstrated a dramatic reduction. In 2021, 80% of *Klebsiella* isolates were MDR, whereas in 2024, this proportion had dropped to 8.3%. The z-statistic of 7.27 and the corresponding *p*-value of approximately 3.54 × 10^−13^ confirm that this change is not due to chance and represents a statistically significant shift in the resistance landscape.

**Figure 8:**
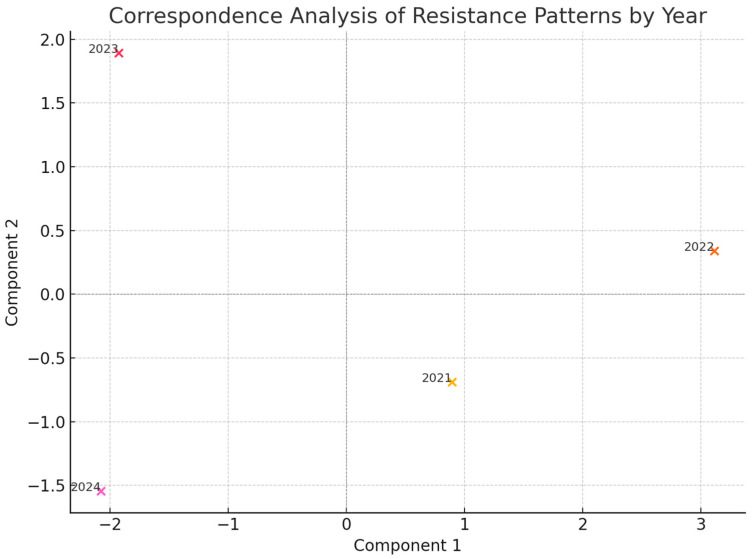
Yearly shifts in Klebsiella pneumoniae resistance profiles mapped via correspondence analysis.

To complement these numerical findings, correspondence analysis using principal component analysis was employed to explore the relationship between years and resistance profiles visually (Figure 8). Each plotted point represents a calendar year, with proximity between points indicating similarity in the resistance distribution. Correspondence analysis demonstrates clustering by resistance pattern. The separation of 2024 indicates the dominance of XDR/PDR phenotypes and a decline in MDR isolates compared to previous years.

**Figure 9:**
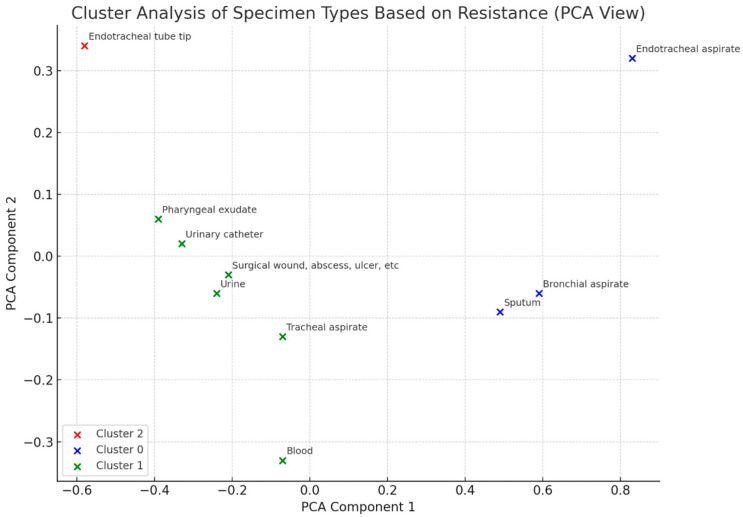
Cluster-based grouping of specimen types by resistance profiles.

In parallel, a cluster analysis was conducted to examine how different specimen types were grouped according to their resistance profiles (Figure 9). This revealed three major clusters, indicating that certain types of samples tend to share similar resistance patterns. For instance, tracheal aspirates and urine specimens clustered based on their high representation of resistant isolates, suggesting common risk factors or clinical pathways. Such clustering can inform targeted infection control interventions by prioritizing specimen types with higher resistance burdens.

Taken together, these analyses present a cohesive narrative of declining MDR prevalence, increasing phenotypic complexity, and clear trends related to specimen resistance. This multifaceted statistical approach strengthens the conclusion that resistance patterns are not only changing over time but are also shaped by the anatomical and procedural context of infection.

## 3. Discussion

This four-year surveillance study provides an assessment of the evolving antimicrobial resistance patterns in *Klebsiella pneumoniae* isolates from ICU patients at a tertiary care center in Romania. The findings indicate a dynamic resistance landscape, marked by a notable decline in MDR phenotypes and a parallel surge in XDR and PDR strains. These shifts carry significant clinical and epidemiological implications, particularly in critical care environments where infection control and empirical therapy require ongoing adjustments.

Over the four-year surveillance period, the distribution of *Klebsiella pneumoniae* resistance patterns among ICU isolates revealed significant shifts. Initially, in 2021 and 2022, multidrug-resistant strains were predominant, with MDR prevalence reaching 80% in 2021 and peaking in 2022. This was followed by a marked decline in MDR prevalence to 8.3% by 2024, suggesting a potentially positive effect of local antimicrobial stewardship and infection prevention initiatives. However, this decrease in MDR was counterbalanced by a rise in resistance, as XDR and even PDR phenotypes emerged and expanded during 2023 and 2024. Rather than reflecting an actual reduction in the resistance burden, these trends highlight an evolution toward increasingly difficult-to-treat strains, likely driven by selective pressures from the continued use of broad-spectrum antibiotics, especially among critically ill ICU patients receiving prolonged courses of therapy. This shift from MDR to XDR and PDR highlights how the ongoing use and misuse of antibiotics lead to the development of resistant organisms. As this cycle continues, bacteria may evolve resistance to an ever-broader range of antibiotics, advancing from MDR to XDR and even PDR. The persistent detection of ESBL-producing and non-resistant isolates across all study years further illustrates the dynamic and heterogeneous evolution of *Klebsiella pneumoniae* resistance [24,25,26,27,28].

Specimen-type stratification added valuable information to help understand *Klebsiella pneumoniae* resistance patterns, as they varied substantially across specimen types. Tracheal aspirates are the most significant reservoir for resistant strains, particularly MDR and XDR isolates. This is likely related to the frequent use of mechanical ventilation and prolonged antibiotic therapy in critically ill patients with respiratory compromise, which together create favorable conditions for the colonization and persistence of highly resistant organisms in the lower respiratory tract. Urine specimens also showed a high prevalence of MDR and XDR isolates, reflecting the burden of urinary tract infections commonly associated with indwelling catheter use and extended ICU stays. Blood cultures revealed a concerning number of XDR isolates, highlighting bloodstream infections caused by highly resistant *K. pneumoniae* as a threat. In contrast, sputum showed minimal resistance, potentially due to differences in colonization versus infection dynamics or the stage of clinical presentation. Other specimens, such as wound, abscess, and ulcer samples, displayed a more balanced distribution of MDR and XDR isolates, but in lower absolute numbers, indicating more sporadic patterns of resistance in these sites. Overall, these patterns highlight the respiratory tract as a primary reservoir for resistant Klebsiella while also emphasizing the urinary tract and bloodstream as critical sources of infection in ICU patients [28,29,30,31,32,33,34,35].

The temporal analysis revealed a statistically significant association between the year of isolation and the resistance phenotype, as evidenced by Cramér’s V of 0.43. The correspondence analysis further supported a clear divergence in the resistance profile observed in 2024 compared with preceding years. This temporal stratification highlights the importance of regularly reevaluating empirical treatment protocols, ensuring that antimicrobial therapies are tailored for the most recent resistance patterns. Given the dynamic nature of resistance evolution, treatment guidelines should be regularly updated to reflect current local epidemiological trends, along with guiding informed decisions about appropriate antibiotic therapy, thereby optimizing patient outcomes and helping to slow down the spread of resistance in high-risk settings such as ICUs [14,36,37,38,39].

Our analysis revealed that the origin of the specimen significantly influences the resistance profiles of Klebsiella pneumoniae, with Cramér’s V of 0.24 indicating a moderate association. Though this relationship is weaker than the strong temporal association observed, it highlights how anatomical site-specific factors can shape resistance phenotypes. For example, biofilm formation on medical devices (such as endotracheal tubes in tracheal aspirates or urinary catheters), variable antimicrobial penetration into different tissues, and differences in local immune defenses contribute to site-dependent resistance patterns. These findings underscore the importance of tailoring therapeutic strategies to the type of specimen when treating infections caused by *K. pneumoniae* [40,41,42].

The rising incidence of XDR and PDR phenotypes in this study is consistent with global trends reported by the WHO and ECDC, which highlight *Klebsiella pneumoniae* as a critical-priority pathogen. Our data corroborate concerns that as lower-resistance phenotypes decline, more robust and potentially untreatable strains are developing. This phenomenon necessitates not only prudent antimicrobial use but also the urgent implementation of infection prevention strategies, including stringent hand hygiene, decontamination of invasive devices, and surveillance [43,44,45].

This study has several limitations that need to be acknowledged. Its retrospective design and single-center focus limit the generalizability of the findings. Another limitation is the lack of detailed resistance profiles for specific antibiotic classes within the categories of MDR, XDR, and PDR. Class-specific susceptibility data, particularly for carbapenems, fluoroquinolones, and colistin, were not consistently available throughout the study period, which impeded a more detailed phenotypic analysis. Future research should systematically collect these data to allow for a more precise characterization of resistance patterns. Furthermore, upcoming studies should investigate the molecular mechanisms that contribute to changes in resistance.

## 4. Materials and Methods

Study Design and Setting: This study is a retrospective observational analysis conducted in the intensive care unit of a County Clinical University Emergency Hospital, a tertiary-level referral center located in Romania. The hospital serves a mixed population of medical and surgical ICU patients, functioning as a hub for complex, high-risk cases that often require invasive procedures and extended antimicrobial therapy. The study took place from 1 January 2021 to 31 December 2024, covering a complete four-year surveillance window. The primary aim was to evaluate the evolution of resistance profiles in *Klebsiella pneumoniae* isolates and their association with time and specimen type.

All microbiological records corresponding to ICU patients who tested positive for *Klebsiella pneumoniae* within the defined period were retrieved from the hospital’s electronic laboratory database. The dataset included the following key variables:

- Demographic details, specifically patient age and sex;
- Date of sample collection, used to determine the year of isolation;
- Specimen type, categorized based on the anatomical source (e.g., tracheal aspirate, blood, urine, catheter tip, and wound exudate);
- Antimicrobial resistance phenotype of the isolate;
- Type of infection, where explicitly noted in the clinical record (e.g., ventilator-associated pneumonia and bloodstream infection).

To maintain analytical consistency and avoid inflation of case counts, each isolate was counted only once per infection episode. Repeat isolates from the same patient were excluded unless they represented a new infection event.

Microbiological Testing: Bacterial species were identified using traditional biochemical tests, including triple sugar iron (TSI), motility indole urea (MIU), Simmons citrate, Gram staining, and methylene blue techniques. Automated identification was conducted using the Vitek 2 Compact analyzer from bioMérieux (376 Chemin de l’Orme, Marcy l’Étoile, France). At our hospital, antimicrobial susceptibility testing is performed automatically with the Vitek 2 system, which employs specialized cards designed for automated processing. Readings are obtained using a turbidimetric method. Broth microdilution was used for colistin testing and to confirm susceptibility patterns in all XDR and PDR isolates, when confirmatory testing was required, particularly for carbapenems and novel β-lactam/β-lactamase inhibitors, as automated systems can be unreliable in these settings.

Antimicrobial resistance profiles were categorized into defined phenotypes, and we included the antibiotics used to define resistance phenotypes (Table 1):

- MDR (Multidrug-Resistant): Resistance to at least one agent in three or more antimicrobial classes.
- XDR (Extensively Drug-Resistant): Resistance to all but one or two available antimicrobial categories.
- PDR (Pandrug-Resistant): Resistance to all antibiotics tested.
- ESBL (Extended-Spectrum Beta-Lactamase Producers): Confirmed via phenotypic methods according to standard testing algorithms.
- No Resistance Pattern: Isolates exhibiting full or near-complete susceptibility to tested agents.

Only isolates confirmed to be *Klebsiella pneumoniae* were included. Mixed cultures or polymicrobial results were excluded unless *K. pneumoniae* was the dominant pathogen.

Statistical Analysis: All statistical analyses were conducted using IBM SPSS Statistics for Windows, Version 29.0 (IBM Corp., Armonk, NY, USA). The following analytical workflow was applied:

Chi-square tests of independence were used to evaluate the relationship between year/specimen type and resistance categories.

Cramér’s V statistic was used to measure the strength of the association for categorical data in the Chi-square analyses.

Standardized residuals were computed post hoc to pinpoint cells in the contingency tables with statistically significant deviations from the expected frequencies.

Logistic regression models were used to predict the probability of an MDR phenotype based on independent variables such as year and specimen type.

Trend analyses included logistic regression over time and two-proportion z-tests comparing MDR prevalence between selected years (e.g., 2021 vs. 2024).

Correspondence analysis, using principal component analysis, was employed to visualize year-wise resistance profile clustering.

K-means clustering was used to group specimen types according to the similarity of their resistance distributions.

Statistical significance was established at *p* < 0.05.

## 5. Conclusions

Our findings indicate a shift in the resistance patterns of ICU-acquired Klebsiella pneumoniae infections, with a rise in XDR and PDR phenotypes replacing MDR strains. Temporal analysis shows evolving resistance profiles, underscoring the necessity for regular updates to empirical treatment guidelines based on current trends. Additionally, stratifying by specimen type identifies critical reservoirs in the respiratory, urinary, and bloodstream systems, emphasizing the influence of site-specific factors on resistance.

## Figures and Tables

**Table 1:** Antibiotics used to test Klebsiella pneumoniae resistance.

Disk Diffusion Antibiogram for Aerobic Bacteria (Kirby–Bauer)
Aztreonam
Ceftazidime/Avibactam
Ceftolozane/Tazobactam
Cefiderocol
**Automated System Antibiogram (Vitek)**
Amikacin
Ampicillin/Sulbactam
Ampicillin ORAL
Aztreonam
Cefepime
Ceftazidime
Ceftriaxone
Chloramphenicol
Cefuroxime ORAL
Gentamicin
Imipenem
Piperacillin/Tazobactam
Tobramycin
Trimethoprim/Sulfamethoxazole
Moxifloxacin
Levofloxacin
Ertapenem
Minocycline
Tetracycline
Tigecycline
Ceftazidime/Avibactam
Ceftolozane/Tazobactam
Cefotaxime
Imipenem/Relebactam
Meropenem/Vaborbactam
Temocillin
Amoxicillin/Clavulanic
Amoxicillin/Clavulanic IV
Cefuroxime
Ciprofloxacin
Meropenem
**Broth Microdilution**
Colistin
**Carbapenemase Detection Test—Enterobacterales**
NDM
KPC
OXA-48-like
IMP
VIM

## Data Availability

Data are contained within this article.
